# Successful Use of Tissue Plasminogen Activator in an Adolescent Male with Pyogenic Liver Abscess

**DOI:** 10.1155/2019/5735312

**Published:** 2019-04-14

**Authors:** Janine Zee-Cheng, Thomas Fox, Sonal Patel, Samer Abu-Sultaneh

**Affiliations:** ^1^Division of Pediatric Hospital Medicine, Section of Pediatric Hospital Medicine, Department of Pediatrics, Indiana University School of Medicine and Riley Hospital for Children at IU Health, Indianapolis, IN, USA; ^2^Division of Pediatric Infectious Disease, Department of Pediatrics, Emory University School of Medicine, USA; ^3^Division of Pediatric Gastroenterology, Department of Pediatrics, Emory University School of Medicine, USA; ^4^Division of Pediatric Critical Care Medicine, Section of Pediatric Critical Care Medicine, Department of Pediatrics, Indiana University School of Medicine and Riley Hospital for Children at IU Health, Indianapolis, IN, USA

## Abstract

Large pyogenic liver abscess is a rare and difficult to treat entity in pediatric patients. Percutaneous drainage rather than open surgical drainage has become more common in recent years, even for large abscesses. Percutaneous drainage can be complicated by catheter obstruction. We present the case of a 16-year-old male presenting with abdominal pain, fever, and chills. He was diagnosed with a 9-centimeter liver abscess. A CT-guided percutaneous drainage was placed. The catheter initially drained well, but then became occluded. Tissue plasminogen activator was instilled into the catheter every 6 hours for a total of five doses, resulting in increased drainage and improved clinical state of the patient. To our knowledge, this is the first reported use of tissue plasminogen activator in pyogenic liver abscess in the pediatric population.

## 1. Introduction

Large pyogenic liver abscesses (PLA) are rare in pediatric patients. Most diagnoses are made in adult patients about 50 years of age with an incidence of 8 to 20 cases per 100,000 admissions [1]. Symptoms of PLAs are nonspecific, and physical examination findings may be subtle, which can present a diagnostic challenge [2]. Despite the low incidence in children of 0.007% to 0.04% of all hospital admissions, PLAs have high mortality rates of 80-100% if left untreated [3].

## 2. Case Presentation

A 16-year-old previously healthy male presented to the emergency department with chills, abdominal pain, worsening nausea, and shortness of breath. He was noted to have increased work of breathing. Abdominal examination revealed mild tenderness in the periumbilical area, but no rebound guarding. His oral temperature was 38.6°C, heart rate 123 beats/min, blood pressure 121/69, and respiratory rate 20 breaths per minute. He was noted to have poor perfusion with capillary refill of 4 seconds, which improved after fluid resuscitation. Complete blood count showed a white blood cell count at 18,300 mm3, platelet count at 78,000/mm3, and hemoglobin of 14.3 g/dL (14-17 g/dL). Blood chemistry demonstrated high blood urea nitrogen at 27 mg/dL, high creatinine at 2.3 mg/dL, high alanine aminotransferase at 442 IU/L, high aspartate aminotransferase at 343 IU/L, and high total bilirubin at 2.8 mg/dL. The patient received one dose of ceftriaxone in the emergency department. He was placed on vancomycin and piperacillin/tazobactam.

**Figure 1:**
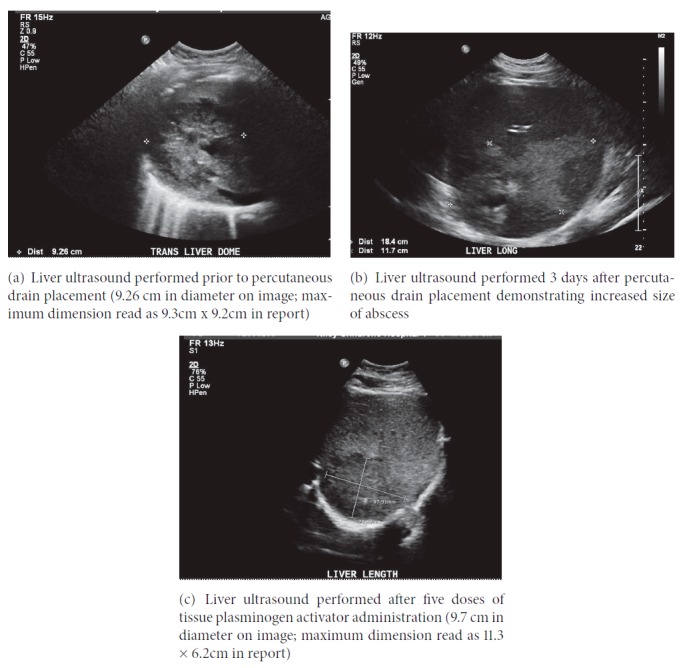


The patient was admitted to pediatric intensive care where he underwent abdominal ultrasound to investigate the acute renal and liver injury. Ultrasound revealed a complex 9.3 x 9.2 cm heterogeneous-appearing mixed soft tissue and cystic lesion within the dome of the right hepatic lobe (Figure 1(a)). After discussion with surgical and infectious disease teams, a CT-guided 10 French pigtail percutaneous drainage catheter (PD) was placed in the abscess and drained purulent brown fluid.

**Figure 2:**
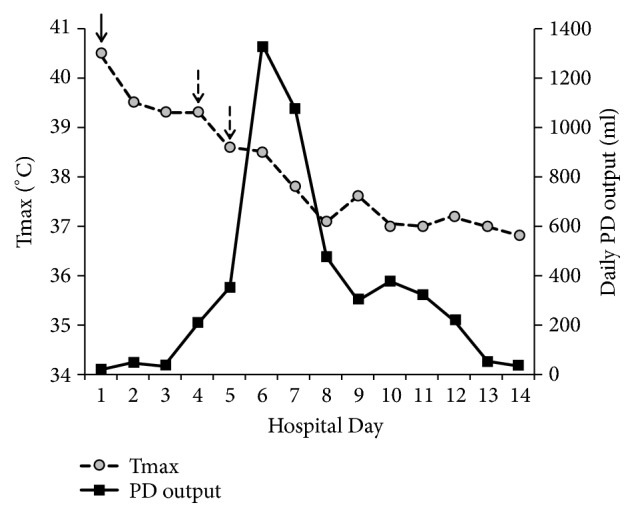
Daily percutaneous drainage (PD) output and maximum temperature (Tmax) during tissue plasminogen activator (tPA) treatment. Solid arrow indicates PD catheter placement and dashed arrows indicate tPA administration days (total of five doses over two days).

The drained fluid grew* Streptococcus anginosus, *which was penicillin susceptible, and antibiotic regimen was changed from piperacillin/tazobactam and vancomycin to ampicillin monotherapy. Metronidazole was added later in his hospital course. Following drainage of the abscess, he became hypotensive, necessitating norepinephrine infusion. He developed respiratory failure requiring noninvasive positive pressure ventilation. Minimal fluid was drained from PD in the first 3 days. Follow-up ultrasound two days after the PD placement demonstrated abscess enlargement (18.4 × 117 cm) (Figure 1(b)). After multispecialty discussion, open surgical drainage was deferred. Instead, tissue plasminogen activator (tPA) was instilled into the PD. Four milligrams tPA was diluted in 50 mL saline, capped for one hour, and then drained to gravity. This was performed every 6 hours for total of five doses. Following instillation of tPA, catheter drainage significantly increased, and the patient's respiratory status and fever curve improved significantly (Figure 2). Follow-up ultrasound after 5 doses of tPA therapy demonstrated a decrease in abscess size to 11.3 × 6.2cm (Figure 1(c)). The patient was discharged home on amoxicillin/clavulanate and metronidazole, with the drain in place, after 14 days of hospitalization. Ultrasound performed eleven days after hospital discharge demonstrated further decrease in size of abscess to 7 × 4.9 cm. The PD was removed 34 days after initial placement.

## 3. Discussion

Pyogenic liver abscesses (PLA) are difficult to diagnose; the triad of fever, jaundice, and right upper quadrant discomfort is present in only 10 percent of patients [4], while fever and right upper quadrant pain without jaundice occurs in just 30% of patients [2]. In developed countries, PLAs occur most frequently in immunocompromised hosts and occur in conjunction with infections in the abdominal cavity, which can aid in diagnosis [5, 6]. In developing countries, PLAs are more common, reported in more than 79 in 100,000 in India [7]. Waghmare et al. studied 34 patients with PLA less than 12 years of age in India, only four of which had predisposing factors for PLA. Three of the patients were managed with intravenous antibiotics only; nine underwent percutaneous needle aspiration with ultrasound guidance; 20 patients required PD. None of the patients required open surgical drainage [8].

Although PLAs are associated with predisposing factors, both pediatric and adult cases have been reported in immunocompetent patients [9–11]. Prompt imaging is important for early diagnosis of liver abscess, if the patient has suggestive symptoms or abnormal laboratory liver function tests, high C-reactive protein, or leukocytosis [12]. Failure to treat PLA is uniformly fatal [13].

Liver abscesses larger than 5 cm in size generally require prompt drainage for resolution of symptoms. It was previously considered a high morbidity disease requiring open surgical drainage, with mortality rates between 9 and 80% [13]. In recent years, there has been a shift from open surgical drainage to PD [14, 15]. Both these treatment options have been demonstrated to effectively drain PLA [16, 17]. Surgical drainage may be preferred in cases in which there is no clinical response after 4-7 days of drainage via catheter; multiple, large, or loculated abscesses; thick-walled abscess with viscous pus; or concurrent intra-abdominal surgical pathology. However, PD is a less invasive, lower-risk procedure and is now a mainstay of treatment [13]. Large abscesses, especially if loculated or containing thick viscid fluid, may fail by PD due to catheter occlusion [18]. Tissue plasminogen activator can be used to improve PD results and avoid open surgical drainage.

Although this patient is physiologically close to adult, he fell outside of the usual age range for PLA in adults and was being treated by pediatric specialists in a pediatric hospital. The high morbidity and mortality rates in untreated pediatric patients necessitate effective treatment. Tissue plasminogen activator has been successfully used to break viscous pus and facilitate drainage of complex parapneumonic empyema in pediatric population [19]. It has been used to facilitate drainage of abdominal abscess in neonatal, pediatric, and adult patients [20, 21]. Criteria for using tPA in parapneumonic effusion include viscous contents with little or no drainage at immediately postdrainage imaging or macroscopically purulent fluid at initial drainage [22]. Adjunctive tPA in the abdominal pelvic abscess cavities has been shown to be safe in adults, even in patients who are being systemically anticoagulated [23]. There currently exist no standardized protocols for treatment with tPA [23], and no prior studies have documented use of tPA in pediatric PLA.

To our knowledge, this is the first reported case of successful usage of tPA to facilitate PD of pediatric liver abscess. The tPA was effective in facilitating the drainage of the PLA in our case that resulted in both clinical and radiological improvements. Caution may be needed when tPA is used as tPA is associated with bleeding; however, it has been safely used in adult patients with intra-abdominal abscess, even in the setting of systemic anticoagulation [23].

## 4. Conclusions

Percutaneous drainage is a safe, effective, less invasive treatment for large pediatric liver abscesses. Tissue plasminogen activator administration can improve drainage in large abscesses, preventing the need for open surgical drainage. More studies are needed to evaluate this treatment option.
